# Health Literacy in Portugal: Results of the Health Literacy Population Survey Project 2019–2021

**DOI:** 10.3390/ijerph19074225

**Published:** 2022-04-01

**Authors:** Miguel Arriaga, Rita Francisco, Paulo Nogueira, Jorge Oliveira, Carlota Silva, Gisele Câmara, Kristine Sørensen, Christina Dietscher, Andreia Costa

**Affiliations:** 1 Divisão de Literacia, Saúde e Bem-Estar, Direção-Geral da Saúde, 1049-005 Lisbon, Portugal; carlota.ribeiro.silva@gmail.com; 2 Católica Research Centre for Psychological—Family and Social Wellbeing (CRC-W), Faculdade de Ciências Humanas, Universidade Católica Portuguesa, 1649-023 Lisbon, Portugal; ritafrancisco@ucp.pt; 3 Instituto de Saúde Ambiental (ISAMB), Faculdade de Medicina, Universidade de Lisboa, 1649-028 Lisboa, Portugal; paulo.nogueira@edu.ulisboa.pt (P.N.); andreiajsilvadacosta@gmail.com (A.C.); 4 Laboratório de Biomatemática, Instituto de Medicina Preventiva e Saúde Pública, Faculdade de Medicina, Universidade de Lisboa, 1649-028 Lisbon, Portugal; 5 Digital Human-Environment and Interaction Lab (HEI-Lab), School of Psychology and Life Sciences, University Lusófona, 1749-024 Lisbon, Portugal; jorge.oliveira@ulusofona.pt; 6 Nursing Research, Innovation and Development Centre of Lisbon (CIDNUR), Nursing School of Lisbon (ESEL), 1990-096 Lisbon, Portugal; giselecpcamara@gmail.com; 7 Global Health Literacy Academy, 8240 Risskov, Denmark; contact@globalhealthliteracyacademy.org; 8 Austrian Ministry of Health, Health Promotion and Disease Prevention, 1010 Vienna, Austria; christina.dietscher@gesundheitsministerium.gv.at

**Keywords:** health literacy, health literacy survey, digital health literacy, navigational health literacy, vaccination health literacy, psychometry

## Abstract

Health literacy entails the knowledge, motivation, and competencies to access, understand, appraise, and apply health information in order to make judgments and decisions in everyday life concerning health care, disease prevention, and health promotion to maintain or improve quality of life throughout the life course. It has become an essential concept in public health. It is considered a modifiable determinant of health decisions, health behaviors, health, and healthcare outcomes. Prior studies suggest highly variable levels of health literacy across European countries. Assessing and monitoring health literacy is critical to support interventions and policies to improve health literacy. This study aimed to describe the process of adaptation to Portugal of the short-form version of the Health Literacy Survey (HLS_19_-Q12) from the Health Literacy Population Survey Project 2019–2021, also establishing the health literacy levels in the Portuguese population. The sample comprised 1247 valid cases. The survey consisted of a brief questionnaire on the determinants of health literacy, plus the HLS_19_-Q12 questionnaire and the specific health literacies packages on digital health literacy, navigational health literacy, and vaccination health literacy. The results suggest that 7 out of 10 people in Portugal (mainland) have high health literacy levels and support the results of other studies concerning the main socioeconomic determinants of general health literacy. Furthermore, the results suggest that “navigation in the health system” tasks are the most challenging tasks regarding specific health literacies. The overall data suggest the HLS_19_-Q12 as a feasible measure to assess health literacy in the Portuguese population. Thus, it can be used in Portugal to assess the population’s needs and monitor and evaluate policies and initiatives to promote health literacy by addressing its societal, environmental, personal, and situational modifiable determinant factors.

## 1. Introduction

The concept of health literacy was introduced in the 1970s, being debated in health care and public health contexts, and has evolved since then as a theoretical concept, leading to the development of different measuring instruments and interventions to promote health literacy [1]. Facing the lack of a consensus about the definition of health literacy or its conceptual dimensions, Sørensen and colleagues, from the Consortium of the European Health Literacy Project (HLS-EU 2009–2012), conducted a study capturing evidence-based dimensions of health literacy and proposed an integrated definition and a comprehensive model of health literacy [1]. This study and its products served as a basis for developing a multidimensional, comprehensive questionnaire to measure health literacy in the general population, the HLS-EU-Q47, containing 47 items across 12 subdomains [2]. The HLS-EU-Q47, supplemented with an additional section with 39 items referring to determinants and consequences outlined in the conceptual model (HLS-EU-Q86), was used to conduct the first comparative European Health Literacy Survey (HLS-EU) in 2011 [3].

This first HLS-EU was conducted in eight of the 27 European Union countries: Austria, Bulgaria, Germany, Greece, Ireland, the Netherlands, Poland, and Spain, comprising 8000 participants. The study disclosed worrying and uneven levels of health literacy (general) between and within countries. More than 10% of the total surveyed population had an inadequate level of health literacy, and this proportion varied between 1.8% in the Netherlands and 26.9% in Bulgaria. Almost 50% of citizens showed limited health literacy (inadequate or problematic), ranging from 28.7% in the Netherlands to more than 62.1% in Bulgaria. Population subgroups characterized by financial deprivation, low social status, low education, or old age had higher proportions of limited health literacy [3].

Three years later, Portugal conducted a survey using the HLS-EU-Q47. The results demonstrated that the general health literacy levels of the Portuguese population (mainland) were very similar to the other countries in the European survey. On the one hand, 11% of respondents showed inadequate health literacy levels, and around 38% showed problematic ones. On the other hand, among the 50% of respondents with adequate health literacy levels, only 8.6% showed excellent ones [4].

As stated by HLS-EU Consortium, monitoring health literacy can support professional and political decision making to improve health literacy to benefit the population’s health [3]. For this purpose, under the umbrella of the World Health Organization’s European Health Information Initiative, the Action Network on Measuring Population and Organizational Health Literacy (M-POHL) was founded in 2018. The Health Literacy Population Survey Project 2019–2021 (HLS_19_) is the M-POHL’s first project. It aims to collect comparative data on the population’s health literacy in as many member states of the World Health Organization European Region as possible [5].

HLS_19_ builds upon the first European Health Literacy Survey and the World Health Organization’s booklet “Health literacy: the solid facts”, and is the result of a systematic and comprehensive review of scientific and experiential evidence on health literacy. Thus, it considers as its conceptual framework the integrative concept developed by the European Health Literacy Consortium [5]: “Health literacy is closely linked to literacy and entails the knowledge, motivation and competencies to access, understand, appraise, and apply health information in order to make judgments and take decisions in everyday life concerning health care, disease prevention and health promotion to maintain or improve quality of life throughout the life course” [1] (p. 3). This integrative concept is based on a previous study that proposes a comprehensive health literacy model that considers 12 domains of health literacy, proximal and distal factors that impact health literacy, as well as the pathways linking health literacy to health outcomes [1].

The generic HLS_19_ questionnaire was built on the HLS-EU-Q47 instrument. Taking the perceived length implementation of HLS-EU-Q47 into account, the new version of HLS_19_ also considers two short forms of 12 and 16 items (HLS_19_-Q12 and HLS_19_-Q16, respectively), besides the HLS_19_-Q47. All these three versions allow evaluation of the general health literacy (HL), three specific domains of health literacy—health promotion, disease prevention, and healthcare (or managing disease)—as well as four aspects of health-related information management, to find/access, understand, evaluate, and apply information relevant for health [5]. 

In Portugal, we opted to adapt the core health literacy measurement questions (HLS_19_-Q12 version) and three optional packages—Digital Health Literacy (HLS_19_-DIGI), Navigational Health Literacy (HLS_19_-NAV), and Vaccination Health Literacy (HLS_19_-VAC). Up to 20 countries have already committed themselves to participating in HLS_19_, and Portugal is one of them [5]. Therefore, as part of the M-POHL and complying with what was suggested in the Portuguese Health Literacy Action Plan [6], the Directorate-General of Health conducted a new assessment of the health literacy of the Portuguese population (mainland), whose results will contribute to developing policies and initiatives to promote health literacy, monitoring their implementation, and comparing results with other European countries. This paper presents and discusses the results of this study, which aimed to:

- translate and adapt HLS_19_ tools (HLS_19_-Q12 and three optional packages) for assessing personal health literacy in Portugal;
- explore psychometric characteristics of the Portuguese HLS_19_ Questionnaire (HLS_19_-Q12);
- establish health literacy levels in the Portuguese population;
- measure new topics on health literacy, specifically digital health literacy (HL-DIGI), navigational health literacy (HL-NAV), and vaccination health literacy (HL-VAC) in the Portuguese population;
- explore associations between health literacy levels and some health literacy correlates (determinants and consequences) in the Portuguese population.

## 2. Materials and Methods

The study population consisted of the inhabitants of Portugal (mainland), above 16 years old, with telephone or mobile phone numbers. The sampling procedures were based on stratified random sampling, with replacement, according to the distribution of the Portuguese population on the following variables: (i) number of residents by NUTS III (NUTS—Nomenclature of Territorial Units for Statistics), (ii) gender, and (iii) large age groups.

Data collection was conducted according to the format of Computer Assisted Telephone Interviews for individuals living in Portugal (mainland) through a telephone line or mobile phone. It was conducted between 10 December 2020 and 13 January 2021. The average time of the calls was 20 min.

To achieve 1247 valid interviews, a total number of 6749 phone calls were made. From these, 4492 were not answered, and 702 individuals refused to participate. Of the 1555 surveys, 30 were not successfully validated (percentage higher than 20% of data incomplete). Therefore, the participation rate in this survey was 69%.

The questionnaire used in this study comprised the core health literacy measurement questions (HLS_19_-Q12), along with 31 core correlates items (relevant health information; determinants; support from others; biometric variables; health habits; health perception; and use of emergency services) and three optional packages (Digital Health Literacy,—16 additional items; Navigational Health Literacy,—12 additional items; and Vaccination Health Literacy,—14 additional items), as shown in Table 1.

The original version of the questionnaire was independently translated to Portuguese by two translators with high proficiency in English. A committee then evaluated these initial translated versions to reach a consensus version. This consensus version was then considered by different experts from the health field and academia in two focus groups to assess the overall understanding of the questions, including laypeople with different socioeconomic backgrounds. Finally, the feedback from these focus groups was incorporated into the consensus version to reach the final version of the Portuguese HLS19 Questionnaire.

The health literacy levels were standardized to a range between 0 and 100. The cut-offs for categorizing health literacy were based on the following positions: below 50; between 50 and 66.66; between 66.67 and 83.33; above 83.34. These cut-offs allowed the definition of the same categories as in the HLS-EU study: “inadequate” and “problematic” (low health literacy), “adequate” and “excellent” (high health literacy).

The statistical analysis was conducted in three different steps. First, a descriptive analysis was performed. Second, we aimed at finding associations between the main determinants of health literacy and the general health literacy (HL) accessed by the Portuguese version of the HLS_19_-Q12. Finally, we aimed at testing the Portuguese version of the HLS_19_-Q12 for reliability, convergent validity, and factorial validity by calculating the Cronbach’s alpha for each scale, establishing the convergent validity by correlating the scores from these scales, and testing factorial validity with confirmatory factor analysis to obtain main fit indices for the Portuguese version of the HLS_19_-Q12. The alpha level for these analyses was set to 0.05. This analysis was conducted using the software IBM SPSS Statistics (IBM, Armonk, NY, USA).

## 3. Results

### 3.1. Descriptive Statistics

#### 3.1.1. Correlate Items

The sample comprised 643 female (52%) and 604 male (48%) participants, with a mean age of 46 (SD = 16.7) ranging from 16 to 87 years old. From the total sample (*n* = 1247), most participants were born in Portugal (*n* = 1148; 92.1%), followed by Brazil (*n* = 28; 2.2%) and Angola (*n* = 22; 1.8%). The other participants were born in other diverse countries. A similar trend was found for the nationality of parents.

Regarding formal education, this was analyzed according to the International Standard Classification of Education (ISCED), showing that the most general education level was upper secondary education—ISCED 3 (*n* = 380; 30.5%), followed by a bachelor or equivalent degree—ISCED 6 (*n* = 272; 21.8%) and primary education—ISCED 1 (*n* = 208; 16.7%).

Concerning employment status, 680 participants were employed (54.6%), whereas 211 were retired (16.9%), 136 were students or trainees (10.9%), and 104 were unemployed (8.3%). Most of these participants reported not being trained in a healthcare profession (*n* = 869; 69.7%).

In the item related to socioeconomic status, the participants were asked to rate their perceived socioeconomic level in Portuguese society on a scale of 1 to 10. A plurality of participants chose the median level 5 (*n* = 358; 28.7%), followed by level 6 (*n* = 325; 26.1%) and by level 4 (*n* = 210; 16.8%), a proportion that decreased for the most extreme higher and lower positions.

Concerning income sufficiency, most participants reported it being “easy to pay all the expenses at the end of the month” (*n* = 667; 53.5%), 439 participants said “difficult” (35.2%), 68 participants reported “very difficult” (5.5%), and 40 participants reported “very easy” (3.2%).

Regarding the difficulty to afford medication if needed, most participants reported it being “easy” to afford medications (*n* = 809; 64.9%), and 87 participants reported being “very easy” (7.0%), but 266 participants reported “difficult” (21.3%), and 28 reported “very difficult” (2.2%). The pattern of response was similar for the items related to the difficulty to afford medical examinations, with 770 reporting “easy” (61.7%), 75 reporting “very easy” (6.0%), 310 reporting “difficult” (24.9%), and 32 reporting “very difficult” (2.6%).

Table 2 summarizes the sociodemographic data along with the income sufficiency of the sample.

Regarding the variables related to the support from others, most participants reported that they “may count on 3 to 5 close persons in case of serious personal problems”. This option was chosen by 511 (41.0%) of the participants, followed by the option of “1 to 2 persons” (*n* = 397; 31.9%) and “6 or more” (*n* = 317; 25.4%). Most participants also answered that those who are close show “a lot of concern and interest” in the things they do in a general way (*n* = 935; 75.9%), followed by “some concern and interest” (*n* = 268; 21.6%). Finally, most participants reported that is “easy” to obtain practical help from neighbors (*n* = 598; 49.1%), followed by “difficult” (*n* = 374; 30.7%).

As for health habits, most participants reported not smoking (*n* = 1006; 80.7%), a large percentage of respondents (43.7%) reported drinking alcoholic beverages “occasionally”, and 29.3% reported “never” consuming alcohol. Furthermore, 17.6% of respondents consume alcoholic drinks 6 or 7 days a week.

On the other hand, regarding physical activity, 29.6% of respondents reported “never” being physically active for 30 min or more, 24.3% reported “occasionally” being physically active for 30 min or more, and 13.6% reported being physically active for 30 min or more “one or two days a week”. Only 24.2% of respondents reported practicing physical activity “four to seven days a week”.

In addition, most respondents (*n* = 856; 68.6%) reported consuming fruits and vegetables 6 or 7 days a week, but 4.7% of respondents (*n* = 59) reported “never” or “occasionally” consuming.

A total of 637 participants (41.1%) reported “good” health regarding self-health perception, but 402 (32.2%) reported “fair” health. In the same way, most participants reported not having a long-term illness or health problem (*n* = 803; 64.7%), and 70.8% (*n* = 685) considered that health problems did not limit their usual activities.

The use of health services was evaluated according to six independent variables. The frequency analysis conducted for each variable showed that most participants reported: “not having used the emergency services in the previous 24 months” (*n* = 756; 60.6%); “not having consulted a general practitioner or family doctor in the previous 12 months” (*n* = 514; 41.2%); “not having consulted a medical or surgical specialist in the previous 12 months” (*n* = 672; 53.9%); “not having been in a hospital as an inpatient in the previous 12 months” (*n* = 1142; 91.6%); and “not having been in a hospital as a day patient in the previous 12 months” (*n* = 1158; 92.9%). In addition, most participants reported “not having been absent from work for health problems in the last 12 months” (*n* = 1077; 86.4%).

#### 3.1.2. General Health Literacy

**Figure 1:**
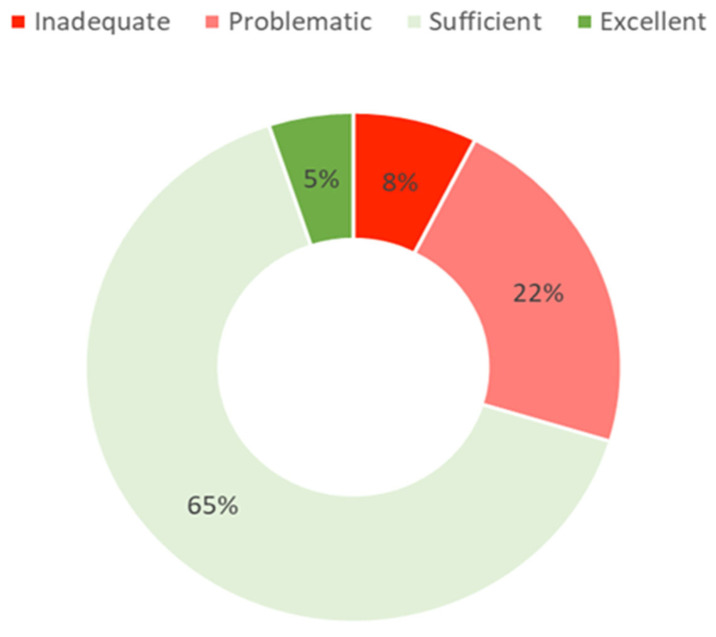
Distribution of the general health literacy levels of the Portuguese HLS_19_ sample [7].

General health literacy (HL) was calculated using the sum of the scores of the core health literacy measurement items (HLS_19_-Q12) that were standardized to a 0–100 scale. The categories were created from the cut-offs provided above. The descriptive analysis shows a mean HL score of 63.8 (SD = 11.5). The frequency analysis of HL categories shows a higher proportion of participants with high HL (i.e., corresponding to “adequate” and “excellent” HL) than with low HL (i.e., corresponding to “inadequate” and “problematic” HL). Most cases were classified as “adequate” HL (65%), followed by “problematic” HL (22%) and “inadequate” HL (7.5%). Only 5% of the cases were classified with “excellent” HL (Figure 1).

#### 3.1.3. Dimensions of Health Literacy

**Figure 2:**
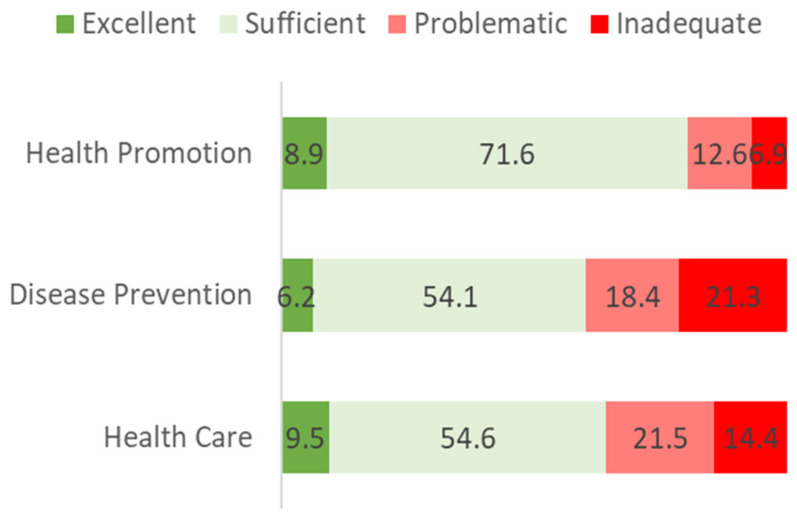
Dimensions of health literacy (%)[7].

The dimensions of health literacy were calculated using the HLS_19_-Q12 instrument, with the grouping of items corresponding to health promotion, disease prevention, and health care. According to the data (Figure 2), it seems more challenging for people to process information related to disease prevention, as the percentage of inadequate levels was 21.3% compared to 14.4% for health care and 6.9% for health promotion. The health-promotion dimension is one in which individuals have higher levels of health literacy, revealing a percentage of sufficient levels of 71.6% compared to 54.6% for health care and 54.1% for disease prevention.

#### 3.1.4. Processing Health-Related Information

**Figure 3:**
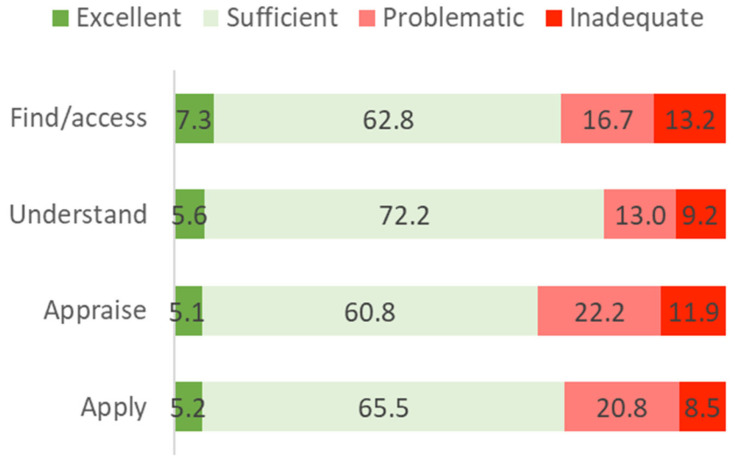
Processing health-related information [7].

The frequency analysis of the ability to process health-related information shows a higher percentage of groups corresponding to high health literacy levels. Understanding health-related information is considered the most accessible aspect. Sufficient levels were reported by 72.2% of participants compared to 65.5% for applying, 62.8% for finding/accessing, and 60.8% for appraising health-related information. On the other hand, to appraise that information is the most complex aspect, where the problematic levels reported in 22.2% exceeded the remaining categories (Figure 3).

#### 3.1.5. Health Literacy Optional Packages

The optional packages used in this study comprised the Digital Health Literacy (HLS_19_-DIGI), the Navigational Health Literacy (HLS_19_-NAV), and the Vaccination Health Literacy (HLS_19_-VAC). The scores for the three optional packages were calculated through the sum of their specific items. Each score was converted to a scale of 0–100 and then recoded into four categories (inadequate, problematic, adequate and excellent) just as it was done for general health literacy (HL).

The descriptive analysis of HL-DIGI showed a mean score of 73.9 (SD = 28.7). The frequency analysis of the category distribution revealed a similar distribution compared to general (HL). However, in this case, the overall proportion of “low” digital health literacy (i.e., corresponding to “inadequate” and “problematic” HL-DIGI) was more pronounced, with a total of 52.7% of the sample, and 47.3% showed an “adequate” level of HL-DIGI.

The descriptive analysis of HL-NAV showed a mean level of 64.2 (SD = 32.4). The frequency analysis of the category distribution revealed lower HL-NAV levels than HL-DIGI. Most participants showed “low” HL-NAV, where “problematic” HL-NAV accounted for 21.5% and “inadequate” HL-NAV for 44%.

The HLS_19_-VAC module was divided into three subdimensions, namely: knowledge on vaccination (three items) with scores between 0 and 3 measured on an ordinal scale; confidence in vaccinations (four items) with scores between 1 and 4; and vaccination health literacy—HL-VAC (four items) with scores between 0 and 100, in which higher scores describe better knowledge, confidence, and health literacy levels on vaccination. One of the HLS_19_-VAC items does not load on any of these dimensions (OP-VAC4 —“Vaccination is compatible with my religious beliefs”). The data regarding knowledge on vaccination showed that most of the participants scored at the highest level (59.1%). As for confidence in vaccinations, the descriptive analysis revealed a mean level of 3.3 (SD = 0.42) and for HL-VAC, a mean level of 87.1 (SD = 22.5). In HL-VAC, 8.8% of participants showed “excellent” levels, and 62.4% showed “sufficient” levels. On the other hand, 15.1% of participants showed “problematic” levels, and 13.7% showed “inadequate” levels.

**Figure 4:**
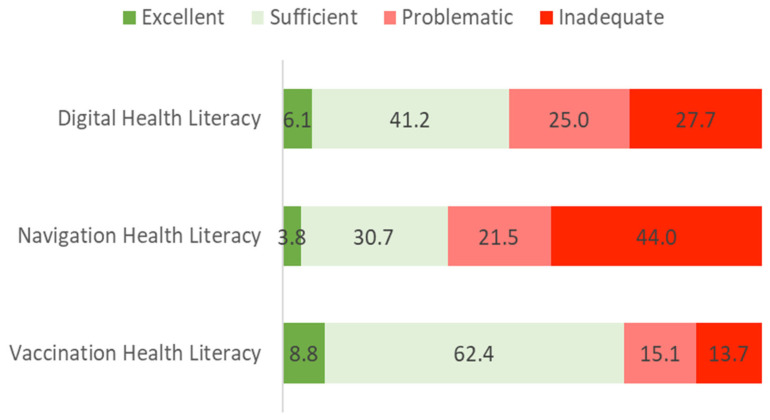
Distribution of the digital health literacy, navigational health literacy, and vaccination health literacy levels of the Portuguese HLS_19_ sample [7].

Figure 4 illustrates frequency analysis of the category distribution of HL-DIGI, HL-NAV, and HL-VAC levels of participants.

### 3.2. Inference Statistics

#### 3.2.1. Determinants of Health Literacy

The potential influence of the determinants of health literacy on general health literacy (HL) was tested using bivariate and multivariable modelling. The first approach explored the possible relationships between HL and each variable. This analysis indicated statistically significant associations in the expected direction between HL and age (*p* < 0.001), gender (*p* < 0.01), economic capacity to pay for medical examinations (*p* < 0.001), education (*p* < 0.001), professional status (*p* < 0.001), health profession (*p* < 0.01), ability to pay expenses (*p* < 0.001), perceived socioeconomic status (*p* < 0.001), and economic capacity to buy medicines (*p* < 0.001).

The second approach studied these influences by testing their respective main effects to identify the simultaneous impact of these determinants of health literacy. This model demonstrated a significant joint effect (F(34) = 9.460; MSE = 3265.499; *p* < 0.001) of age, gender, economic capacity to pay for medical examinations, education, and professional status, indicating higher levels of literacy in the younger age groups, males, with greater economic capacity, in people with higher levels of schooling, and employees. Variables such as the health profession, economic capacity to buy medicines, ability to pay expenses, and perceived socioeconomic status did not contribute significantly (*p* > 0.05) to this statistical model after adjusting for the other factors studied.

#### 3.2.2. Correlations between HL, HL-DIGI, HL-NAV and HL-VAC

The correlations between these measures were conducted using Pearson product-moment correlation coefficient. These results indicated statistically significant correlations between general health literacy and digital health literacy (r = 0.549; *p* < 0.001), navigational health literacy (r = 0.530; *p* < 0.001), and vaccination health literacy (r = 0.358; *p* < 0.001).

#### 3.2.3. Internal Consistency for HLS_19_-Q12 and Optional Packages

The internal consistency for the HLS_19_-Q12 scale was calculated with Cronbach’s alpha. The Cronbach’s alpha was 0.902, which describes a good internal consistency for the scale in this 12-item version. Inter-item correlations were appropriate, with a mean inter-item correlation score of 0.442, ranging between 0.279 and 0.648. None of the items would increase internal consistency if deleted from the scale.

The same analysis was conducted for the items comprising the Digital Health Literacy optional package. The Cronbach’s alpha was 0.749, which revealed a satisfactory internal consistency score. However, the inter-item correlations were higher for HLS_19_-DIGI than for HLS_19_-Q12, suggesting a high redundancy of this scale’s items. Mean inter-item correlation was 0.556, ranging between 0.364 and 0.818. None of the items would increase internal consistency if deleted from the scale.

Regarding Navigational Health Literacy optional package, the consistency analysis through Cronbach’s alpha revealed a higher consistency value than the other scales. The Cronbach’s alpha was 0.939. Likewise, this increased value may reveal redundancy in items comprising this scale, supported by a mean inter-item correlation of 0.566, which ranges between 0.380 and 0.825.

As for Vaccination Health Literacy, the internal consistency through Cronbach alpha level revealed an adequate level for vaccination health literacy (0.718). The mean inter-item correlation was 0.402, ranging between 0.242 and 0.598. Regarding the other subdimensions, the Cronbach’s scores were 0.892 for the subdimension confidence in vaccinations, and 0.655 for the subdimension knowledge on vaccination. The mean inter-item correlations were 0.673 (ranging from 0.564 to 0.859) and 0.388 (ranging from 0.316 to 0.464) for confidence in vaccinations and knowledge on vaccination, respectively.

### 3.3. Factorial Structure for HLS_19_-Q12

#### 3.3.1. Exploratory Factor Analysis (EFA)

The factorial structure for HLS_19_-Q12 was explored based on a two-step procedure. First, the database was split into two different datasets to conduct an exploratory factor analysis, followed by a confirmatory factor analysis. The results from both steps are described below.

The file was split into two different files based on a randomly generated variable using a random number generator in SPSS. The median value for this distribution was calculated to divide the overall sample (*n* = 1247) into two datasets, one with 623 and the other with 624 cases, fulfilling the minimum sample size criteria for conducting these procedures [8]. Multicollinearity between items was also assessed as a requisite for Exploratory Factorial Analysis (EFA) through Variance Inflation Factor (VIF). The VIF values calculated for each item showed values within the normal range, i.e., below VIF < 10 [9].

The first procedure was based on EFA. This procedure was conducted in JASP (University of Amsterdam, Amsterdam, The Netherlands) based on the principal axis factoring method for factor extraction. According to the Kaiser Rule, extraction was based on Eigenvalues higher than 1 [10]. Missing values were pairwise excluded.

**Figure 5:**
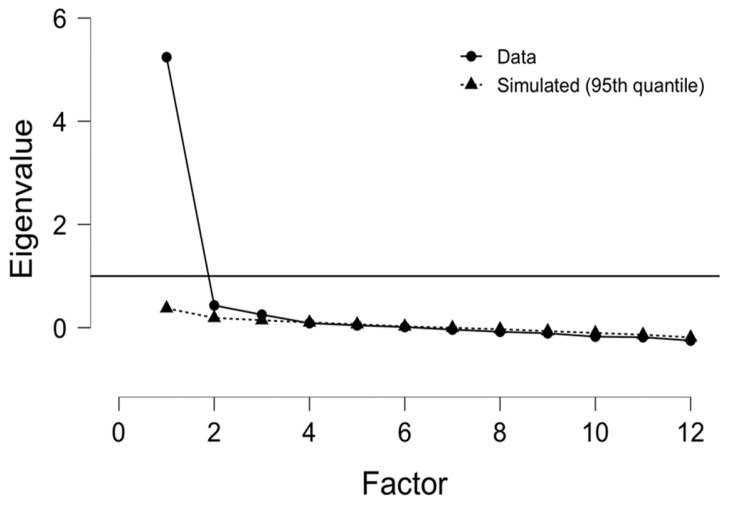
Scree plot for exploratory factor analysis.

The results from EFA to this dataset showed adequate Kaiser–Meyer–Olkin (KMO = 0.925) for measuring sampling adequacy. The Bartlett’s Test of Sphericity supported the validity of this analysis by indicating a statistically significant test for the Chi-square distribution (χ^2^(66) = 327.351; *p* < 0.001), whereas the Chi-square test for the overall model was also significant (χ^2^(54) = 371.999; *p* < 0.001). According to the Kaiser Rule for factor extraction, the best solution that fits these data is a one-dimensional solution shown in the Scree plot (Figure 5).

The factorial structure’s factor loadings showed adequate loadings for each item (all items > 0.500), as shown in Table 3.

#### 3.3.2. Confirmatory Factor Analysis

The factorial structure for HLS19-Q12 was tested using Confirmatory Factor Analysis (CFA). This analysis was conducted in JASP software to test a one-dimensional model. This solution was drawn from prior literature suggesting the acceptable goodness-of-fit indexes using CFA for the one-dimensional structure of HLS-EU-Q12 [11].

**Figure 6:**
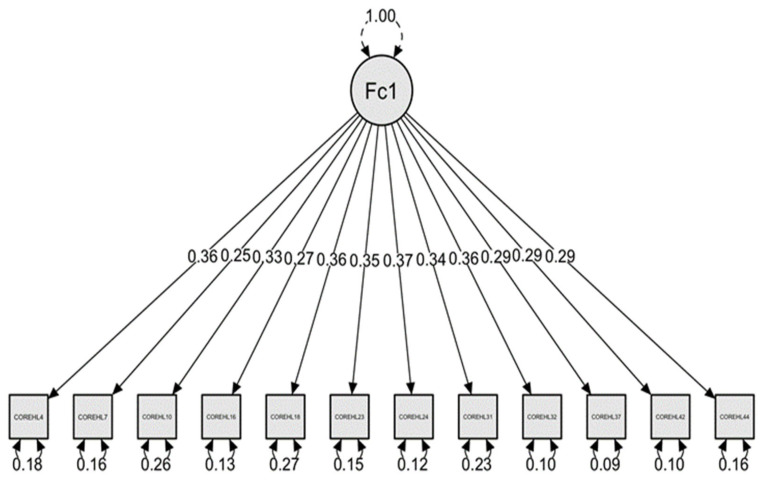
HLS_19_-Q12 model from Confirmatory Factor Analysis. Fc1 is factor one of the scale in the confirmatory factor analysis.

The CFA was conducted with the algorithm from MPlus with the Maximum Likelihood estimator and based on the Robust method. This analysis revealed an overall good-of-fit based on fit indices, according to the following information: RMSEA (root mean square error of approximation) = 0.079 (LI 90 = 0.069, LS 90 = 0.089), CFI (Comparative Fit Index) = 0.922; TLI (Tucker–Lewis Index) = 0.904; GFI (Goodness-of-fit index) = 0.995; NFI (Bentler–Bonnett Normed Fit Index) = 0.904, IFI (Bollen’s Incremental Fit Index) = 0.922; PNFI (Parsimony Normed Fit of Index) = 0.740. According to Bentler [12], RMSEA less than 0.08, GFI, NFI, and IFI greater than 0.90, and PNFI greater than 0.50 reflect adequate structural models. The CFI above 0.90 is considered adequate [13]. Figure 6 depicts the resulting structural model.

## 4. Discussion

This study aimed to validate and culturally adapt the original version of the HLS_19_-Q12 to Portugal, assess the health literacy levels of the mainland Portuguese population based on a representative sample, and assess new topics on health literacy, namely, digital health literacy, navigational health literacy, and vaccination health literacy.

Most participants presented high levels of HL, corresponding to 65% of “adequate” and 5% of “excellent” levels of HL. Concerning the lower levels of HL, 7.5% of participants presented “inadequate”, and 22% “problematic” levels of HL. The results suggest that 7 out of 10 people have high levels of health literacy, representing an increase in higher levels of health literacy compared with previous studies which used the original and more extended version of this questionnaire (HLS-EU-Q47) [4]. However, these comparisons need to be cautiously interpreted since HLS_19_-Q12 is a newly adapted tool to measure population health literacy, making a direct comparison with previous studies unfeasible.

Considering the different dimensions of HL—health promotion, disease prevention, and health care, participants presented higher levels of health literacy in the health promotion dimension (71.6% and 8.9% sufficient and excellent, respectively), exceeding the levels obtained for the HL index. On the opposite side, processing information related to the disease-prevention dimension of HL is considered the most challenging task for this sample (18.4% and 21.3% problematic and inadequate, respectively). Therefore, it is essential to reinforce health literacy actions focused on disease prevention from very early ages. Meeting the information needs of each target group facilitates active involvement in their own health and the development of health-promoting attitudes, beliefs, and behaviors right during childhood and adolescence [14].

The competence of “understanding information” was associated with the highest levels of HL, with over 75% categorized as having sufficient and excellent levels of health literacy. However, “appraise health-related information” was considered the most challenging aspect for many of our participants (34.1% problematic and inadequate levels of HL). We know that it is not enough to present people with trustworthy information; it is essential to develop actions that promote the ability to interpret, filter, judge, and evaluate the health information that has been accessed. Only this capacity will allow people to communicate and use the information to make good decisions to maintain and improve their health [1].

Regarding specific health literacies, the data revealed poorer levels of navigational health literacy lying below the general health literacy levels, suggesting that navigating the Portuguese health care system is more challenging than the other specific health literacies. According to previous studies in other countries, the demands on patients and users to orient within and navigate health care systems are increasing, as well as the complexity and fragmentation of these systems [15]. As for vaccination health literacy, this domain was measured from the Vaccination Health Literacy module that comprised the four items of the HLS_19_-Q47, which revealed the highest health literacy levels. A possible explanation for these results may be the period in which these data were collected, during the COVID-19 pandemic, which may have motivated people to seek digital information related to vaccination, improving vaccination health literacy levels along with digital health literacy. However, the lack of prior studies regarding specific health literacies such as digital, navigational, and vaccination health literacies in Portugal does not allow firm conclusions about whether these levels improved during the COVID-19 pandemic.

Furthermore, the analysis of the psychometric properties showed appropriate scores for internal consistency for each of these subscales, highlighting the usefulness of these instruments for assessing and monitoring specific health literacies in further research.

The results of the bivariate statistical analysis between the determinants of health literacy and the general health literacy index (HL) started to point to the results of other studies, which ultimately led to the generally accepted conclusion that low HL disproportionately affects the most vulnerable populations [16,17,18]. That is true for the statistically significant associations found between HL and gender, age groups, professional status, education levels, and economic capacity to afford medical examination if needed. However, no statistically significant associations were found between HL and nationality as happened in some other studies that found lower HL in migrant populations [16].

The multivariate analysis results are similar to those from the first European Health Literacy Survey that pointed to a combined effect of financial deprivation, social status, education, age, and gender in HL [3]. However, it is interesting to analyze the different associations found for some variables used to assess socioeconomic status, such as the “economic capacity to afford medical examination if needed”, the “self-perceived socioeconomic level”, the “capacity to pay all the expenses at the end of the month”, and the “capacity to afford medication if needed”. From the results, we can hypothesize that, at least in Portugal, the first one may be better to assess socioeconomic status, as it is the only variable for which a statistically significant association with HL was shown. Different results were found in the first European Health Literacy Survey [3].

The psychometric properties were tested for validity and reliability of the HLS_19_-Q12 scale. The correlations among the HLS_19_-Q12 measure with the optional packages for HL-DIGI, HL-NAV, and HL-VAC were conducted to contribute to convergent validity of the general health literacy score with the digital, navigational, and vaccination health literacy scores, revealing statistically significant associations between these dimensions. The factor structure of the HLS_19_-Q12 measure was also explored using EFA to understand whether the data are adjustable to a one-dimensional factor structure. The results from the EFA and a further CFA suggested that the most suitable solution is a one-dimensional factor solution. These results are aligned with those from prior studies [11], suggesting the HLS_19_-Q12 measure as a feasible measure to assess health literacy.

The main limitation of this study is the period when these data were collected, which may have contributed to bias some of the results, explicitly concerning digital and vaccination-specific health literacies. Moreover, the lack of previous assessments of specific health literacies in Portugal has also limited the comparison between our data and normative data for digital, navigational, and vaccination health literacies. Furthermore, the fact that HLS_19_-Q12 is a new measure of general health literacy does not allow us to compare our results with previous studies about the levels of health literacy in the Portuguese population, since a different measure was used [4]. The understanding of the sample characteristics of non-responders was also not possible to ascertain because no information was collected for non-responders. Finally, the cross-sectional study design does not allow causal interpretations concerning health literacy determinants.

Future studies using HLS_19_-Q12 may allow monitoring of health literacy levels in the Portuguese population, also enabling comparisons with other countries. HLS_19_-Q12 can assess needs, and monitor and evaluate policies and initiatives to promote health literacy at local, regional, and national levels in Portugal.

Considering the comprehensive model behind HLS_19_-Q12 development, actions should focus on modifiable determinants of health literacy, moving beyond individual skills development approaches. Policies and initiatives aiming to improve health literacy must reflect the relational nature of the concept, which involves the interaction of settings, people, and professionals of many sectors, and propose more integrative intersectoral approaches that also address literacy-related barriers to information, services, and care and promote supportive systems (e.g., health-literate settings; health literacy-friendly organizations; health-promoting schools; plain language; supportive environments for consumers; increasing capacity building on health literacy) [16,19].

## 5. Conclusions

The results of HLS_19_ in Portugal suggest a strategic focus on interventions that improve health literacy for health promotion, disease prevention, health care, and health system navigation. Our results also suggest a social gradient for health literacy. Women, older age groups of individuals, and people with lower economic capacity, lower levels of schooling, and the unemployed are at risk of low HL in Portugal. Particular attention should be given to these population groups regarding actions to promote HL.

Considering the comprehensive model behind HLS_19_-Q12 development, this study supports the need to move beyond individual skills development when planning policies and initiatives aiming at the population’s health literacy improvement, proposing integrated and intersectoral perspectives to address literacy-related barriers to information, services, and care.

The comparison of the HLS_19_-PT results with the results of the Health Literacy Survey from the other European countries will be essential to analyze the performance of the HLS_19_-Q12. For now, this study suggests the HLS_19_-Q12 as a feasible screening measure to assess health literacy in the Portuguese population. Continuous and regular monitoring of health literacy, its domains, and associated factors is essential to evaluate policies and practices and propose specific approaches that fit groups of individuals and health literacy domains, reducing inequities in the populations’ health and gaps and inefficiencies in health promotion initiatives and health systems.

## Figures and Tables

**Table 1:** Overview of HLS_19_ and Optional packages.

HLS_19_ Core	HLS_19_-Q12	HL Measurement	HL Correlates
		Short Version with 12 Items for Measuring General Health Literacy	Mandatory Correlates for General HL Measurement (31 Items) Such as Age, Education, Socioeconomic Status, etc.
Optional packages	Digital Health Literacy (HLS_19_-DIGI)	Sub-scale with 16 items for measuring digital health literacy	
	Navigational Health Literacy (HLS_19_-NAV)	Sub-scale with 12 items for measuring navigational health literacy	
	Vaccination Health Literacy (HLS_19_-VAC)	Sub-scale with 14 items for measuring vaccination health literacy	

**Table 2:** Sociodemographic and income sufficiency data.

	Frequency (*n*)	%
Gender		
Male	604	48.4
Female	643	51.6
Formal education		
No formal education or below ISCED 1	98	7.9
ISCED 1 Primary education	208	16.7
ISCED 2 Lower secondary education	199	16.0
ISCED 3 Upper secondary education	380	30.5
ISCED 4 Post-secondary but non-tertiary education	17	1.4
ISCED 5 Short-cycle tertiary education	19	1.5
ISCED 6 Bachelor’s or equivalent level	272	21.8
ISCED 7 Master’s or equivalent level	47	3.8
ISCED 8 Doctoral or equivalent level	7	.6
Employment status		
Employed	680	54.6
Self-employed	81	6.5
Unemployed	104	8.3
Retired	211	16.9
Unable to work due to long-standing health problems	10	.8
Student, trainee	136	10.9
Fulfilling domestic tasks	21	1.7
Compulsory military or civilian service	3	.2
Pay all the expenses at the end of the month		
Very easy	40	3.2
Easy	667	53.5
Difficult	439	35.2
Very difficult	68	5.5
Don’t know/Don’t answer	33	2.6
Afford medication if needed		
Very easy	87	7.0
Easy	809	64.9
Difficult	266	21.3
Very difficult	28	2.2
Don’t know/Don’t answer	57	4.6
Afford medical examination if needed		
Very easy	75	6.0
Easy	770	61.7
Difficult	310	24.9
Very difficult	32	2.6
Don’t know/Don’t answer	60	4.8

**Table 3:** Factor loadings.

	Factor 1
COREHL 4	0.587
COREHL 7	0.599
COREHL 10	0.601
COREHL 16	0.544
COREHL 18	0.672
COREHL 23	0.684
COREHL 24	0.749
COREHL 31	0.635
COREHL 32	0.791
COREHL 37	0.699
COREHL 42	0.720
COREHL 44	0.605

Note. Using promax rotation.

## Data Availability

The data that support the findings of this study are available on request from the corresponding author, Miguel Arriaga. The M-POHL consortium is the owner of the HLS-19 questionnaire rights. More information can be found at https://m-pohl.net/ (accessed on 28 March 2022).

## Abbreviations

HL	General health literacy
HLS_19_-PT	Portuguese Health Literacy Population Survey Project 2019–2021
HLS_19_-Q12	12-item version of the Health Literacy Survey from the Health Literacy Population Survey Project 2019–2021
HLS_19_-Q16	16-item version of the Health Literacy Survey from the Health Literacy Population Survey Project 2019–2021
HLS_19_-Q47	47-item version of the Health Literacy Survey from the Health Literacy Population Survey Project 2019–2021
HLS-EU	European Health Literacy Survey
HLS-EU 2009–2012	European Health Literacy Project
HLS-EU-Q47	European Health Literacy Project 47-item Questionnaire
HLS-EU-Q86	HLS-EU-Q47 supplemented with an additional section with 39 items referring to determinants and consequences used to conduct the first comparative European health literacy survey in 2011
HL-DIGI	Digital health literacy
HL-NAV	Navigational health literacy
HL-VAC	Vaccination health literacy
HLS_19_-DIGI	Digital Health Literacy (instrument)
HLS_19_-NAV	Navigational Health Literacy (instrument)
HLS_19_-VAC	Vaccination Health Literacy (instrument)
ISCED	International Standard Classification of Education
M-POHL	Action Network on Measuring Population and Organizational Health Literacy
HLS_19_	Health Literacy Population Survey Project 2019–2021
EFA	Exploratory factor analysis
VIF	Variance Inflation Factor
CFA	Confirmatory Factor Analysis
